# Hypo- and Hypermorphic *FOXC1* Mutations in Dominant Glaucoma: Transactivation and Phenotypic Variability

**DOI:** 10.1371/journal.pone.0119272

**Published:** 2015-03-18

**Authors:** Cristina Medina-Trillo, Francisco Sánchez-Sánchez, José-Daniel Aroca-Aguilar, Jesús-José Ferre-Fernández, Laura Morales, Carmen-Dora Méndez-Hernández, Fiona Blanco-Kelly, Carmen Ayuso, Julián García-Feijoo, Julio Escribano

**Affiliations:** 1 Área de Genética, Facultad de Medicina, Universidad de Castilla-La Mancha, Albacete, Spain; Instituto de Investigación en Discapacidades Neurológicas (IDINE), Universidad de Castilla-La Mancha, Albacete, Spain; 2 Cooperative Research Network on Age-Related Ocular Pathology, Visual and Life Quality, Instituto de Salud Carlos III, Madrid, Spain; 3 Servicio de Oftalmología, Hospital Clínico San Carlos, Madrid, Spain; Instituto de Investigación Sanitaria del Hospital Clínico San Carlos, Madrid, Spain; 4 Servicio de Genética, Instituto de Investigación Sanitaria-Fundación Jiménez Díaz (IIS-FJD), Madrid, Spain; Centro de Investigación Biomédica en Red de Enfermedades Raras (CIBERER), Madrid, Spain; NIDCR/NIH, UNITED STATES

## Abstract

Dominant glaucoma, a heterogeneous, infrequent and irreversible optic neuropathy, is often associated with elevated intraocular pressure and early-onset. The role of *FOXC1* in this type of glaucoma was investigated in twelve Spanish probands via nucleotide variation screening of its proximal promoter and unique exon. Functional evaluations of the identified variants included analyses of the transcriptional activity, protein stability, DNA binding ability and subcellular localization. Four different mutations that were identified in four probands (33.3%) were associated with remarkable phenotypic variability and were functionally classified as either hypermorphic (p.Y47X, p.Q106X and p.G447_G448insDG) or hypomorphic (p.I126S) alleles. To the best of our knowledge, three of the variants are novel (p.Y47X, p.I126S and p.G447_G448insDG) and, in addition, hypermorphic *FOXC1* mutations are reported herein for the first time. The presence of an intact N-terminal activation domain in the truncated proteins p.Y47X and p.Q106X may underlie their associated transactivation hyperactivity by a gain-of-function mechanism involving dysregulated protein-protein interactions. Similarly, altered molecular interactions may also lead to increased p.G447_G448insDG activity. In contrast, the partial loss-of-function associated with p.I126S was due to impaired protein stability, DNA binding, protein phosphorylation and subcellular distribution. These results support that moderate and variable *FOXC1* transactivation changes are associated with moderate goniodysgenesis, dominant glaucoma and remarkable phenotypic variability.

## Introduction

Glaucoma is a highly heterogeneous, irreversible and progressive blinding disease produced by the death of the retinal ganglion cells, which results in the degeneration of the optic nerve. Raised intraocular pressure (IOP) is the primary risk factor for developing glaucoma. Most glaucoma cases behave as a late-onset complex disease and a minority of cases show an early- onset and follow simple Mendelian inheritance. Dominant transmission is typically observed in juvenile open-angle glaucoma (JOAG; MIM#137750), as well as in some cases of both adult-onset primary open-angle glaucoma (POAG; MIIM# 137760) and primary congenital glaucoma (PCG; MIM# 231300) [1, 2]. PCG is caused by developmental abnormalities in the anterior segment of the eye, which is required for aqueous humour drainage, and manifests clinically in the neonatal or infantile period, generally before the age of 3. Moreover, late congenital glaucoma cases (LCG, i.e., glaucoma diagnosed over 3 years of age with abnormal gonioscopy of the anterior segment and dominant inheritance) have also been described [3]. So far two primary genes, *CYP1B1* [4] (MIM# 601771) and *LTBP2* [5] (MIM# 602091), have been identified in recessive PCG. Loss-of-function *CYP1B1* mutations are the predominant known genetic cause of this type of glaucoma in different populations [6–8]. *MYOCILIN* (*MYOC*, MIM# 601652) [9, 10] and *FORKHEAD BOX C1* (*FOXC1* MIM# 601090) alterations have also been found in a small number of PCG patients [3, 10]. JOAG presents with an early age of onset, usually between 10 and 35 years. Although at least five loci have been identified for this type of dominant glaucoma, only one disease-causing gene (*MYOC*) has been identified thus far [11]. *MYOC* mutations are present in approximately 10% of all JOAG cases [12]. Finally, nine dominant adult-onset POAG loci have been reported, but only three genes related to this type of glaucoma have been identified, *OPTN* (MIM# 602432) [13], *ASB10* (MIM# 615054) [14] and *WDR36* (MIM# 609669) [15].

*FOXC1* lies in the 6p25 forkhead cluster (*FOXC1/FOXF2/FOXQ1*) and mutations in this gene cause a spectrum of autosomal dominant anterior eye segment defects, including Axenfeld-Rieger syndrome type 3 (ARS; MIM#602482), causing an increased risk for glaucoma and varying degrees of iris or extra-ocular abnormalities [16]. This gene encodes a member of the FOX class of transcription factors, which are involved, among other processes, in the regulation of craniofacial, cardiovascular and ocular development [17]. A characteristic and conserved 110-amino acid DNA-binding domain, known as the forkhead domain (FHD), is present in the protein. *FOXC1* is expressed in mesoderm and neural-crest-derived cells, including the cells in the anterior segment of the eye, and the periocular mesenchyme and mesenchymal cells that have migrated into the eye [18, 19]. The transcriptional activity of this phosphoprotein is regulated by N- and C-terminal activation domains [20]. To date, more than fifty different missense, nonsense, and frameshift *FOXC1* mutations have been identified, with the majority affecting the forkhead domain [21]. These mutations reduce the FOXC1 transactivation ability [22], and none have been described to increase FOXC1 activity. However, increased activity is likely to be associated with duplications in *the gene*, *which has been* observed in various types of anterior segment disorders and glaucoma [23]. It has been shown that patients diagnosed with Axenfeld-Rieger malformation who carry *FOXC1* duplications have a more severe prognosis for glaucoma development than patients with *FOXC1* mutations [24].

To our knowledge, this is the first study to identify hypermorphic *FOXC1* mutations, and to show that moderate and variable residual FOXC1 activity levels are involved in dominant glaucoma, and may contribute to the phenotypic variability that is present in this disease.

## Materials and Methods

### Ethics statement

The study and informed consent procedures were approved by the Ethics Committee for Human Research of the Hospital Clínico San Carlos, Madrid (Spain), and followed the tenets of the Declaration of Helsinki. Informed written consent was obtained from all of the participants and was recorded by staff involved in the study.

### Subjects

Twelve unrelated probands affected by dominant glaucoma were included in this study. All subjects were clinically evaluated by glaucoma specialists. The ophthalmic examination included slit lamp biomicroscopy, gonioscopy, biometry, intraocular pressure (IOP) measurement and ophthalmoscopy. The PCG clinical diagnosis included at least two of the following clinical features; increased corneal diameter (>12 mm), along with elevated IOP (>21 mmHg or >16 mmHg under general anaesthesia with sevoflurane) and/or Haab’s striae, corneal edema and optic disc changes, usually before the age of 3. The preoperative PCG diagnosis was based on early clinical signs and symptoms (photophobia, blepharospasm, tearing, corneal edema and corneal enlargement). Patients over 3 years of age who were diagnosed with abnormal gonioscopy of the anterior segment (high iris insertion and absence of an angle recess), were considered to be LCG cases. Cases diagnosed between 10 and 35 years of age or over the age of 40, were classified as JOAG and POAG, respectively. All of the following criteria were required for either the JOAG or POAG diagnosis: open anterior chamber angle, IOP > 21 mm Hg, characteristic optic disc changes (e.g., vertical cup-to-disc ratio > 0.4, thin or notched neuroretinal rim or disc haemorrhage), and characteristic visual field changes. The visual field alterations were classified as previously described [25]. Secondary glaucoma probands were excluded from the study. In children, the intraocular pressure was measured using the Perkins applanation tonometer (Clement Clarke MK2, Harlow, UK), generally under sevoflurane anaesthesia; and as soon as they were sufficiently anesthetized to check the intraocular pressure (during the first 10 min after induction and measured again immediately before the children wake up when they breathe air). Older and more cooperative children were assessed using a slit lamp for tonometry with a Goldmann applanation tonometer.

### Mutation screening

Genomic DNA was extracted from the peripheral blood, using the *QIAamp DNA Blood Mini Kit* (Qiagen). The DNA sequence variation analyses were carried out using automatic Sanger sequencing. The promoter (nucleotides −1 to −875), the translated region and the 5'- and 3'-untranslated regions (UTRs) of *FOXC1* were amplified via PCR using the primers described in S1 Fig. and S1 Table. The mutations were confirmed in two independent PCR amplifications by DNA sequencing.

### Site directed mutagenesis and cloning of the identified *FOXC1* mutations

**Three *FOXC1* mutations (**p.Y47X, p.Q106X and p.I126S) and the control *FOXC1* mutation p.I126M **were obtained via site-directed mutagenesis using** the QuickChange Site-directed Mutagenesis Kit (Stratagene), with the primers and PCR conditions indicated in the S1 Materials and Methods and S2 Table. The p.G447_G448insDG variant was directly amplified from the genomic DNA of the carriers using primers P1 and P2 (S1 Materials and Methods), and were cloned into the *EcoRI/BamHI* restriction sites of the pcDNA3.1(-) vector. All of the recombinant FOXC1 versions were transiently expressed in human embryonic kidney 293T (HEK-293T) cells purchased from the American Type Culture Collection (ATCC Rockville, MD) as previously described [26, 27] and indicated in the S1 Materials and Methods. Transient plasmid transfections were carried out using 50–500 ng of plasmid DNA using the Superfect Transfection Reagent (Qiagen), according to the manufacturer’s instructions.

### Transcriptional activity assays

The FOXC1 transactivation assays were performed using the Luciferase Assay System (Promega) according to the manufacturer’s instructions. A 600-bp fragment of the human *CXCR4* gene promoter, which contains one FOX-binding element (FBE) [28], was cloned into the *Nhe*I/*Nco*I restriction sites of the pGL3-basic vector (Promega) via directional PCR using normal human genomic DNA as a template and the following primers: 5’TCTG**GCTAGC**GCGCGGGGAATGGCGTTGG3’ and 5’CT**CCATGG**TAACCGCTGGTTCTCCAG3’ (the *Nhe*I and *Nco*I sequences are indicated with bold letters, respectively). The monkey kidney derived COS7 cell line has been widely used in functional analyses of *FOXC1* mutations associated with human ocular phenotypes [29, 30]. In this study, we employed the human embryonic kidney 293T cell line (HEK-293T). HEK-293T cells in 24-well tissue culture plates (2.5x10^5^ cells/well) were transfected with 500 ng of the FOXC1 expression vector, along with 50 ng of the recombinant pGL3-basic-*CXCR4* luciferase reporter, and 50 ng of the pMirTarget vector (Origene), which expresses red fluorescent protein (RFP) as a transfection control. The total amount of transfected DNA was equalized to the empty vector. The transactivation assays were performed 48 h after the transfection. The cells were harvested and assayed for firefly luciferase activity as previously indicated. FOXC1, RFP and LDH were detected by Western blot as the expression, transfection and loading controls, respectively. The RFP and LDH bands were quantitated via densitometry (n≥3) and significant differences were analyzed using the *t*-test.

### Protein stability and half-life calculation

The protein stability was studied via western blot of the transfected cells incubated with cycloheximide (300 μg/ml) at different times, as previously reported [31]. The FOXC1 protein levels were determined by densitometry and the relative amounts at the different times after cycloheximide treatment were expressed as a percentage of the levels at time 0 h. At least three independent assays for each variant were performed. The transfection efficiency was assessed by co-transfecting the cDNAs encoding the different mutants (500 ng) with the pMirTarget vector (50 ng), which encodes RFP. The fluorescent protein was detected via western blotting. The loaded samples were normalized for total protein content using Bradford reagent (Pierce). FOXC1 decay follows a first-order kinetics. The slope of the decay line was calculated using standard linear regression, and the protein half-life was determined as previously described [32].

### Nuclear protein extraction and Electrophoretic Mobility–Shift Assay (EMSA)

The nuclear extracts of the HEK-293T cells expressing recombinant FOXC1 proteins were prepared as previously reported [22] and as briefly described in the S1 Materials and Methods. An EMSA was performed using the LightShift EMSA Optimization and Control Kit (Thermo Scientific) according to the manufacturer's protocol and asdescribed in the S1 Materials and Methods.

### FOXC1 treatment with calf intestinal alkaline phosphatase

The phosphorylation status of the FOXC1 protein expressed in the HEK-293T cells was analyzed by calf intestinal alkaline phosphatase (CIP) treatment of the nuclear extracts. The nuclear extracts were incubated with either 5 units of CIP, 11 μ**m** sodium vanadate (NaVO_3_), or both for 2 h at 37°C. The recombinant proteins were detected via western immunoblot using an anti-myc antibody. The percentage of phosphorylated protein was estimated via densitometry of western blot bands as follows: [upper band volume (phosphorylated FOXC1) x 100]/[upper band volume (phosphorylated FOXC1) + lower band volume (dephosphorylated FOXC1)]. LDH was detected via western blot as the loading control, and was quantitated via densitometry (n≥3), and significant differences were analyzed using the *t*-test.

### Western and dot blotting and antibodies

For the western blot analysis, the nuclear extracts were fractionated using sodium dodecyl sulfate-polyacrylamide gel electrophoresis (SDS-PAGE) using the Mini-PROTEAN III Gel Electrophoresis System (BioRad). A tricine-SDS-PAGE was performed as previously described [33]. The protein content of the samples were normalized using the **Bradford assay**. The gels were subsequently transferred onto Hybond ECL nitrocellulose membranes (Amersham) for the immunodetection, And a dot blot analysis of the non-sense mutants was carried out by spotting the normalized nuclear extracts onto Hybond ECL nitrocellulose membranes in a blot transfer apparatus (Bio-Dot, Bio-Rad). A commercial mouse monoclonal anti-myc (Santa Cruz Biotechnology) antibody was used as the primary antibody, diluted at 1:1000. Horse-radish peroxidase-conjugated antibodies against mouse IgG (Pierce) were diluted to 1:1000–1:4,000. The chemiluminiscence detection was performed using Supersignal Dura Western Blot reagents (Pierce), and luminescent imaging was used for the chemiluminescence detection (LAS3000-mini; Fujifilm, Tokyo, Japan). The ensitometry for the protein band quantitation (FOXC1, LDH and RFP) was performed using the Quantity One 4.1 analysis software package (BioRad) in at least two independent experiments performed in triplicate. Ponceau S (Panreac) staining of the blots prior to the antibody incubation was performed to ensure the integrity of the samples and that equal amounts of the sample were analyzed [34]. As an additional sample loading control, lactate dehydrogenase (LDH) was detected in the cell extracts and culture media using a goat anti-LDH antibody [AB1222, Chemicon, diluted to 1:5000) and an anti-goat IgG horse-radish peroxidase-conjugated antibody (sc-2033, Santa Cruz Biotechnology, diluted to 1:2000). The RFP (transfection control) was detected using a rabbit anti-RFP antibody (#AB233, Evrogen), diluted to 1:5000, and an anti-rabbit IgG horse-radish peroxidase-conjugated antibody (#1858415, Pierce) diluted to 1:1000.

### Immunocytochemistry

The HEK-293T cells were seeded on coverslips placed into 24-well plates, and were transiently transfected with DNA constructs encoding different FOXC1 variants. All of the transfections were performed using the SuperFect Transfection Reagent (Qiagen). After the transfection, the cells were washed once with DPBS and cultured for 24 h. The cells were then fixed with 4% paraformaldehyde for 10 min at room temperature, followed by incubation with phosphate-buffered solution containing 0.2% triton X-100, 10% FBS, and 5% bovine serum albumin for 30 min at room temperature. The recombinant proteins were detected using an anti-myc antibody (Santa Cruz Biotechnology) (at a 1:500 dilution) at 4°C overnight followed by a Cy3-conjugated anti-mouse IgG (Jackson ImmunoResearch Labs,1:1000 dilution) for 2 h at room temperature. Finally, the coverslips were mounted on glass slides using polyvinyl alcohol mounting medium with DABCO (Fluka) containing DAPI (4′,6′-diamidino-2-phenylindole; F6057 SIGMA) and were viewed under a laser scanning confocal microscope (Zeiss LSM 710).

### Bioinformatic analyses

The *In silico* analyses of the sequences of the different *FOXC1* variants were carried out using the programs described in the S1 Materials and Methods. Mutations were named according to RefSeq: NM_001453.2 and using the directions from Mutalyzer (https://humgenprojects.lumc.nl/trac/mutalyzer). The transcription start site and 5’-UTR sequence were defined as previously described [35]. The first nucleotide of the translation initiation site was numbered as nucleotide +1. The novel variants have been submitted to dbSNP (http://www.ncbi.nlm.nih.gov/SNP/).

### Statistical analyses

The statistical comparisons between the groups were performed using either the *t*-test or the one-way analysis of variance (ANOVA). A Bonferroni correction was applied to adjust the tests for multiple comparisons. The data were statistically processed using the SigmaStat 2.0 software (SPSS Science).

## Results

### The identification of rare *FOXC1* mutations

**Fig 1:**
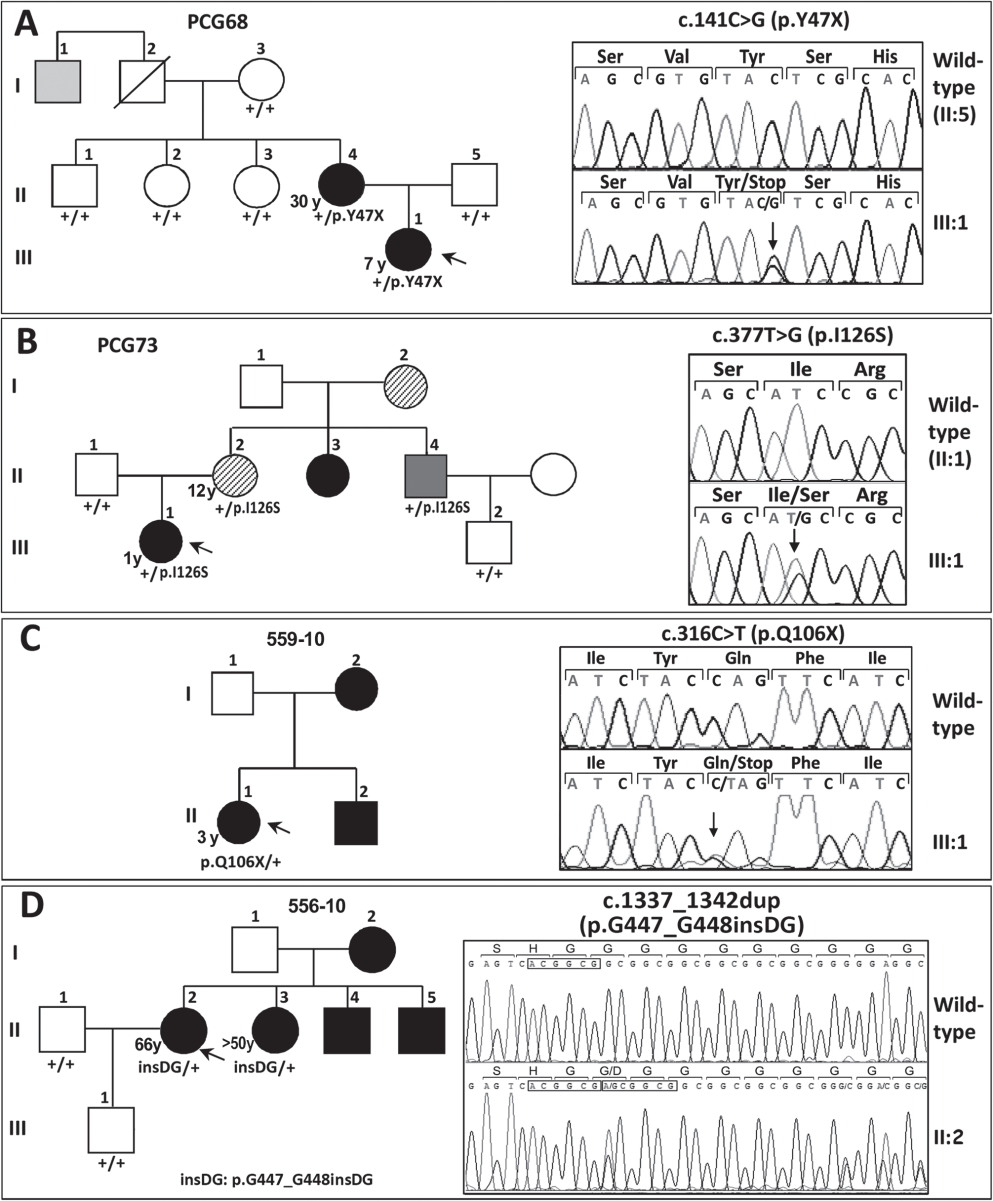
*FOXC1* mutation segregation in families with autosomal dominant glaucoma. **(A, B, C and D)** Pedigrees of the families. The numbers in the inferior left side of the symbols indicate age at diagnosis. The oblique lines represent dead subjects. Black, dark grey and light grey symbols indicate glaucoma, Axenfeld-Rieger anomaly and ocular hypertension, respectively. The arrows in the pedigrees show the index cases. +: wild-type allele. The insets in each panel correspond to the electropherograms of the *FOXC1* mutations identified in each family. To facilitate the comparison, the wild-type sequence is shown above the corresponding mutant sequence. All the mutations were detected in the heterozygous state. Arrows in the electropherograms indicate the location of mutations. Duplicated nucleotides in panel **D** inset are indicated by boxes.

**Fig 2:**
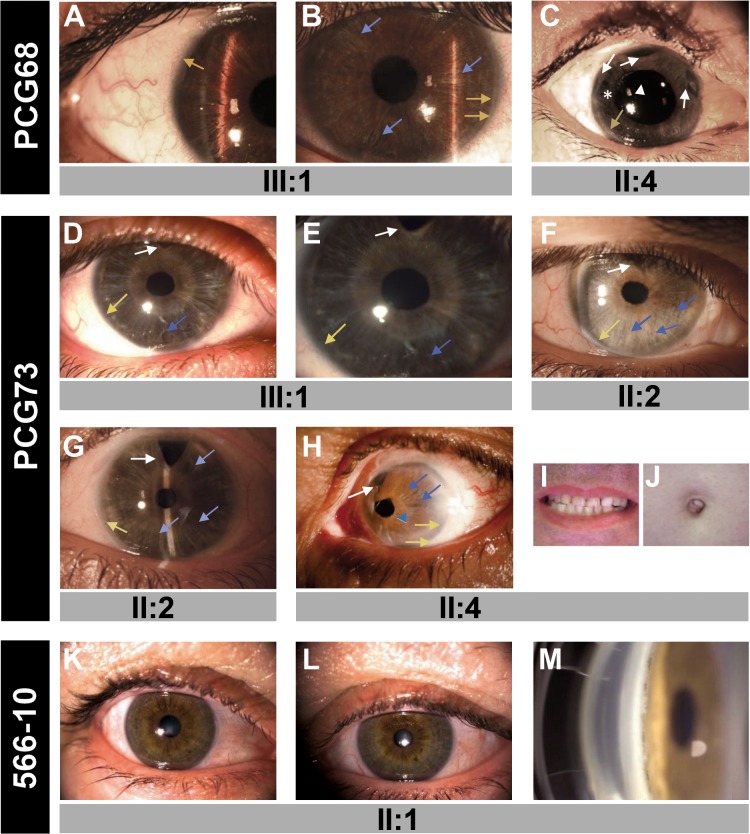
The phenotypes associated with the *FOXC1* mutations identified in this study. **(A)** Right and **(B)** left eye of subject PCG68 III:1. **(C)** Right eye of subject PCG68 II:4. **(D)** Right and **(E)** left eye of proband PCG73 (subject III:1). **(F)** Right and **(G)** left eye of subject III:1. (H) Axenfeld-Rieger anomaly in the left eye of subject PCG73 II:4. **(I)** Normal teeth and **(J)** umbilicus of this patient. **(K)** Right and **(L)** left eye, and **(M)** gonioscopy photography of subject 566–10 II:1. Asterisk: area of iris atrophy secondary to surgery. Blue arrows: areas of thin iris. Blue arrowhead: corectopia. Yellow arrows: posterior embrytoxon. White arrowhead: Ahmed valve tube in anterior chamber. White arrows: surgical iridectomies.

To analyze the role of *FOXC1* mutations in dominant glaucoma, a total of twelve unrelated probands with no mutations in the glaucoma genes *MYOC* and *CYP1B1* were studied. Four of them were affected with PCG, one with LCG, three with JOAG and four with POAG. Four different rare heterozygous sequence variations were identified in four probands (33.3%; two PCGs, one LCG and one POAG; Fig. 1 and Table 1). Three of these variants (p.Y47X, p.I126S and p.G447_G448insDG) were not found in the Emsembl variation database, the Human Gene Mutation Database, GeneCards, OMIM or in pubMed, and were therefore considered to be novel mutations. These mutations were also absent in 200 control chromosomes (data not shown). The mutations p.Y47X and p.I126S segregated with the disease in families PCG68 and PCG73, respectively (Fig. 1A and B). These two families showed remarkable phenotypic variability, particularly, but not exclusively, for the disease onset. Proband PCG68, who was classified as LCG, showed a thin iris stroma and posterior embrytoxon in both eyes and no evident iris hypoplasia, corectopia or systemic alterations (Fig. 2A and B). Her mother, diagnosed with JOAG at the age of 30, also exhibited posterior embryotoxon with no iris attachments or areas of iris atrophy secondary to surgery (Fig. 2C). Generalized stromal thinning, atrophy, corectopia (with the pupil pulled in the direction of the most prominent angle findings), or iris hole formation occurring in the quadrant opposite the site of corectopia, were not observed in these two patients; therefore, according to the 9^th^ Consensus Report of the World Glaucoma Association, the presence of mild iris stroma anomalies in the proband were considered to be compatible with congenital glaucoma [36]. Proband PCG73 was diagnosed with congenital glaucoma and her mother was diagnosed with juvenile glaucoma. Both patients presented thinned iris stromal areas, posterior embryotoxon with no iris attachments, surgical iridectomies in both eyes and normal pupils (Fig. 2D-G). Generalized iris stromal thinning, atrophy, corectopia or iris hole formation were also not detected in these patients. Interestingly, another PCG73 family member, subject II:4, also carried the same *FOXC1* genotype and presented corectopia, generalized iris stromal thinning, which could be secondary to surgery, posterior embryotoxon and surgical iridectomy in his left eye (Fig. 2H). He did not show dental, umbilical (Fig. 2I and J, respectively) or any other systemic malformations, and was diagnosed with Axenfeld-Rieger anomaly (ARA). His right eye was eviscerated at the time of the study. In family 559–10, the proband was the only subject available for genetic analysis, and ocular photographs were not available. She was diagnosed with PCG and carried the nonsense mutation p.Q106X, (Fig. 1C and Table 1). Finally, in the adult-onset glaucoma family (556–10), the proband was diagnosed at the age of 66, and her three siblings manifested glaucoma in their fifties (Fig. 1D and Table 1). Only the index case and her sister consented to the genetic analyses and were found to carry the heterozygous *FOXC1* variant p.G447_G448insDG (Fig. 1D, subjects II:2 and II:3). The proband’s iris and iridocorneal angle were normal (Fig. 2K-M) and a careful clinical examination directed to detected Axenfeld-Rieger alterations did not reveal any other relevant ocular or systemic anomalies. All of the described mutations are associated with bilateral glaucoma, variable iris morphology and a disease onset ranging from 1 to 66 years in the index cases (Table 1). The previously described 3’-UTR variant c.*734A>T (rs35717904) was also identified in probands PCG73 and 556–10 (data not shown). *PITX2* mutations were ruled out in these patients.

**Table 1:** FOXC1 gene variations identified in dominant glaucoma cases and associated clinical features.

Proband or family member	^1^Nucleotide change	Amino acid change	Phenotype/laterality	Age at diagnosis (years)/sex	IOP (mmHg) at diagnosis (OD/OS)	C/D (OD/OI)	^2^Treatment
PCG68 (III:1)	^3^c.141C>G	^3^p.Y47X	LCG/B	7/F	(24/20)	NA	1
PCG68 (II:4)	c.141C>G	p.Y47X	JOAG/B	30/F	30/25	0.8/0.8	5+Surgery
559–10 (II:1)	c.316C>T	p.Q106X	PCG/B	3/F	(25/25.9)	NA	Surgery
PCG73 (III:1)	^3^c.377T>G	^3^p.I126S	PCG/B	1/F	(14/15)	(0.6–0.7/0.8–0.9)	1+Surgery
PCG73 (II:2)	c.377T>G	p.I126S	JOAG/NA	12/F	NA	NA	NA
PCG73 (II:4)	c.377T>G	p.I126S	ARA/B	NA/M	NA	NA	NA
556–10 (II:2)	^3^c.1337_1342dup	^3^p.G447_G448insDG	AOG/B	66/F	(21/20)	NA	1
556–10 (II:3)	c.1337_1342dup	p.G447_G448insDG	AOG/B	>50/F	NA	NA	NA

^1^The variants were present in the heterozygous state;

^2^Number of drugs and/or surgery;

^3^Novel variants;

AOG/ARA/JOAG/LCG/PCG: Adult-onset glaucoma/Axenfeld-Rieger anomaly/juvenile-onset glaucoma/late-onset primary congenital glaucoma/primary congenital glaucoma; B: bilateral; C/D: cup/disk ratio; NA: Not available; OD/OS: right eye/left eye. Mutations were named according to RefSeq: NM_001453.2 and using directions from Mutalyzer (https://humgenprojects.lumc.nl/trac/mutalyzer).

To evaluate the pathogenicity of the identified variants a number of approaches were employed, including bioinformatic analysis, evolutionary conservation of the mutant amino acids or nucleotides and an *in vitro* assessment of transcriptional activity, protein stability, DNA binding ability, phosphorylation status and subcellular localization.

### Bioinformatic analysis and evolutionary conservation of the variants

**Fig 3:**
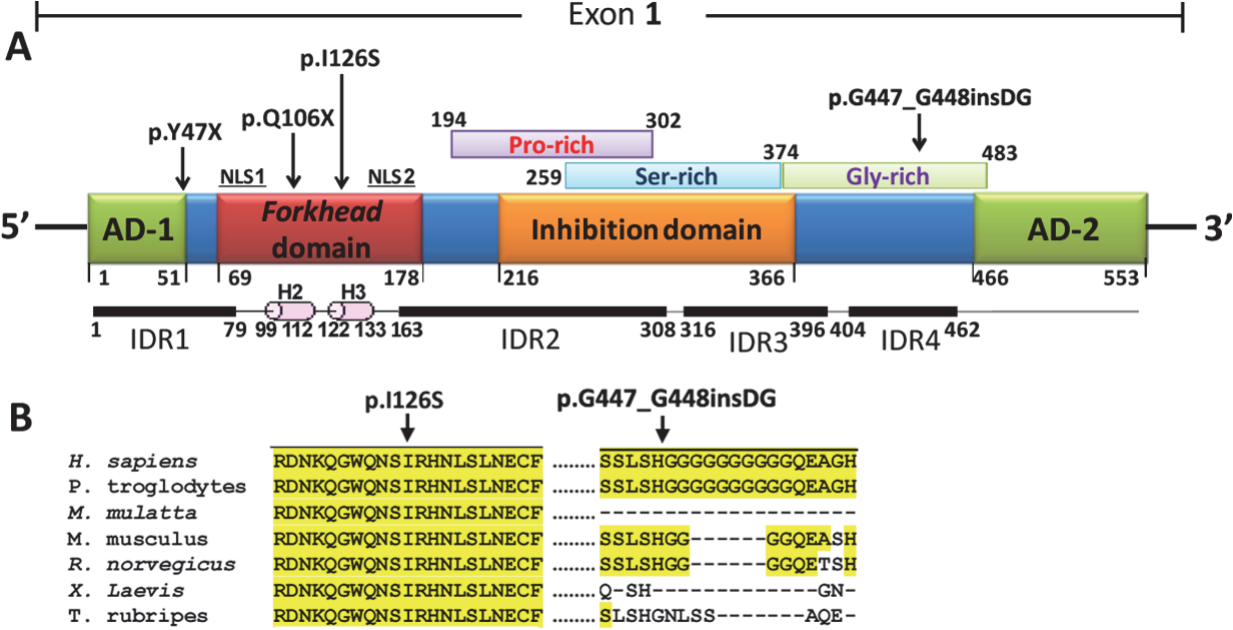
Localization of the *FOXC1* mutations identified in this study. **(A)** Localization of the predicted and previously identified structural domains and motifs of the polypeptide chain. The predicted Pro-, Ser- and Gly-rich regions reported in the Prosite database are indicated over the scheme. Four IDRs predicted with the DisEMBL [50] and Globplot 2 [51] programs, and alpha-helices 2 (H2) and 3 (H3) of the forkhead domain, are indicated below the protein. AD: activation domain. CR: coding region. IDR: intrinsically disordered region. NLS: nuclear localization signal. Arrows note mutated positions. **(B)** Multiple alignments of amino acid or nucleotide sequences affected by mutations. The alignments were carried out with ClustalW [52]. The yellow background indicates the positions where the amino acids are identical.

The analyses of the variants with the programs indicated in the S1 Materials and Methods showed that three coding mutations mapped to activating domain 1 (p.Y47X), alpha helix-2 (p.Q106X) and alpha-helix 3 (p.I126S) of the forkhead DNA binding domain (Fig. 3A). The remaining coding mutation (p.G447_G448insDG) was situated in a Gly-rich sequence that is part of the inferred intrinsically disordered region 4 (IDR4, Fig. 3A). An *in silico* analysis carried out using the SIFT, PolyPhen-2 and Panther programs to evaluate the functional effect of the amino acid substitution p.I126S predicted that it was damaging (SIFT score = 0.01; PolyPhen-2 score = 0.99; Panther Pdeleterious = 0.99). The evolutionary conservation analysis revealed that the amino acid residue I126 is conserved from fish to mammals and that the DG insertion affects a poly-Gly tract that is shared only by humans and chimpanzees (Fig. 3B).

### Transcriptional activity

**Fig 4:**
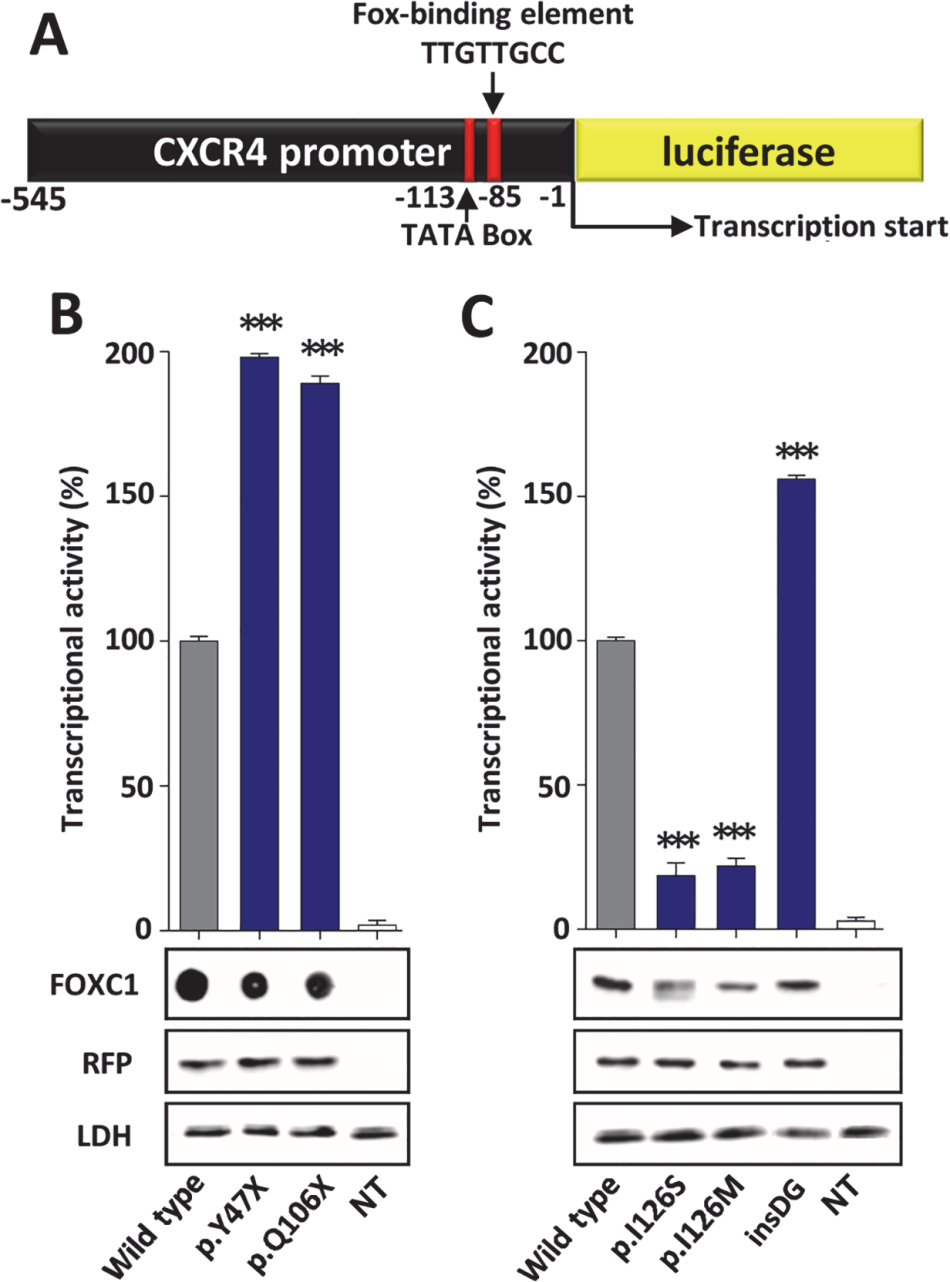
Altered transcriptional activity of the *FOXC1* variants identified in dominant glaucoma patients. **(A)** Scheme of the cDNA construct containing a FOXC1-binding element present in the *CXCR4* promoter fused to the luciferasecoding region. The cDNA was cloned into the PGL3 basic vector. This construct was used as a reporter of transcriptional activity in co-transfection assays with the different *FOXC1* variants and the empty pMirTarget vector, which encodes RFP (transfection efficiency control). The numbers below the scheme correspond to nucleotide positions. cDNA constructs encoding the indicated *FOXC1* variants **(B)** were transiently co-expressed with the reporter cDNA in HEK-293T cells. The transcriptional activity, expressed as a percentage of the luciferase activity of the wild-type protein, was measured as indicated in the Materials and Methods. The protein levels of the different FOXC1 versions present in HEK-293T cells 48 h after transfection were determined by western immunoblot using a monoclonal anti-myc antibody (Santa Cruz), except for the two non-sense mutants (p.Y47X and p.Q106X) which were difficult to detect and quantify by this technique and had to be assesed by dot-blot. Each lane contained 15 μg of total protein obtained from the cell lysates. Transfection efficiency was assessed via western immunoblot using an anti-RFP antibody (Evrogen). The sample loading control, endogenous LDH, was also detected via immunoblot using an anti-LDH antibody (Chemicon). Error bars correspond to the SD of three independent experiments carried out in triplicate. insDG: p.G447_G448insDG. NT: non-transfected cells (negative control). Asterisks indicate statistical significance as compared to the control: p<0.05 (*); p<0.01 (**); p<0.001 (***). Significance was calculated by one-way ANOVA followed by Tukey multiple-comparison test.

The transcriptional activity of the recombinant FOXC1 variants was assessed via a transient co-transfection with the luciferase reporter gene coupled to the *CXCR4* promoter region, which contains one FBE (Fig. 4A). The detection of the truncated proteins p.Y47X and p.Q106X via standard SDS-PAGE and western blotting was prevented due to their small molecular size. To overcome this difficulty, the electrophoresis was carried out in the presence of tricine. Under these conditions, only p.Q106X (but not p.Y47X) could be identified (S2 Fig.). We also observed that p.Q106X and the wild-type protein were transferred into the nitrocellulose membrane with different yields due to their different molecular weights, making the quantification challenging. Because of these limitations, the truncated proteins were finally analyzed via dot-blot. Western or dot-blot analyses of RFP and LDH were used as controls for the transfection efficiency and sample loading, respectively, and revealed no significant differences. Therefore, the observed FOXC1 band intensity variations can be attributed to alterations in the protein stability, which will be shown later. Three mutations (p.Y47X, p.Q106X and p.G447_G448insDG) showed significantly increased transcriptional activity, ranging from approximately 160% to almost 200% of the wild-type protein, and were classified as hypermorphic variants (Fig. 4B and C). In contrast, the activity of the fourth variant (p.I126S) was only 20% of the normal protein and was similar to that of the control mutation p.I126M (Fig. 4C), showing that it is a hypomorphic allele. p.I126M has been identified in patients with ARS and glaucoma [16] and it has been proposed to be a positive control mutant for activity studies in the forkhead family of transcription factors [22].

### Protein stability

**Fig 5:**
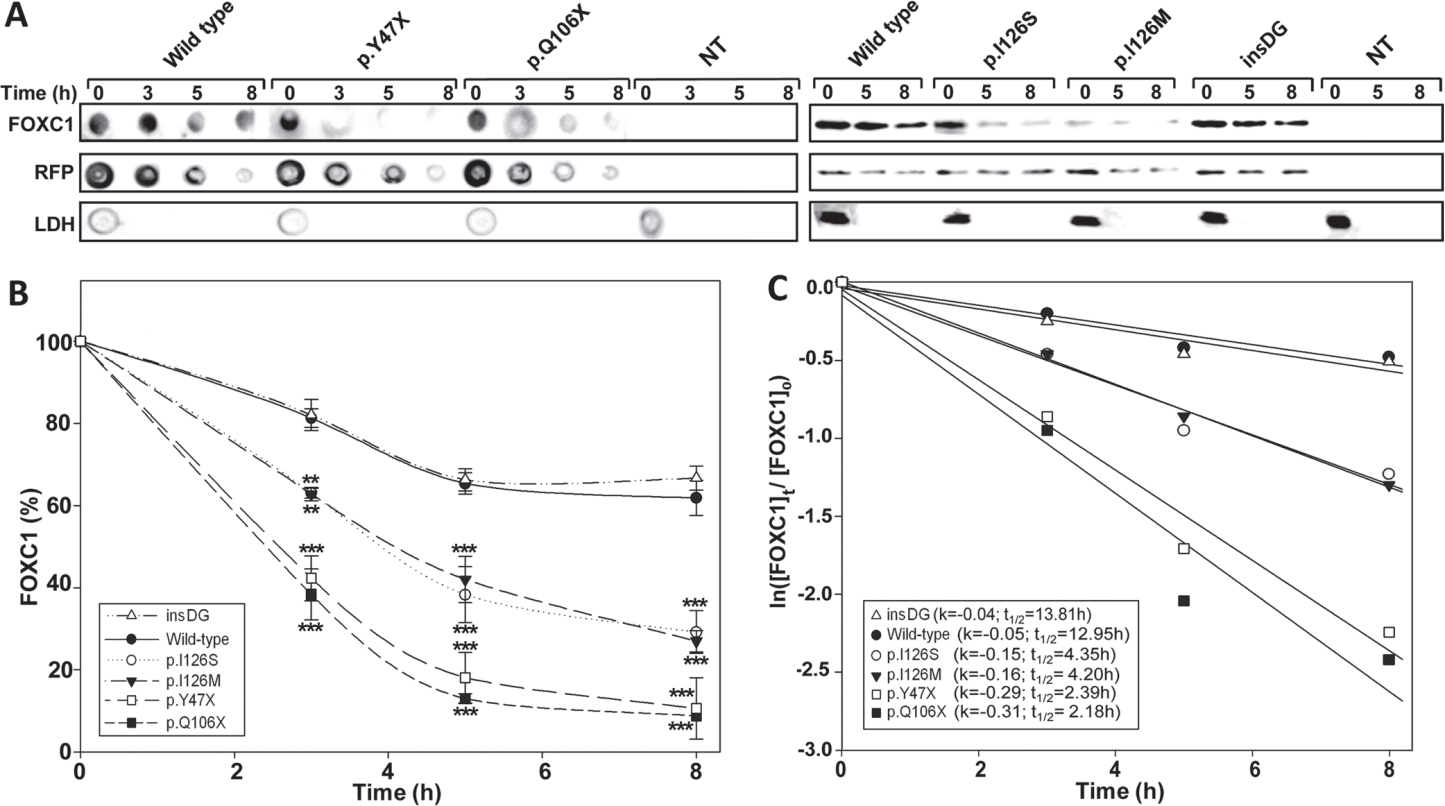
The p.Y47X, p.Q106X and p.I126S glaucoma-associated *FOXC1* mutations decrease protein stability. **(A)** Time course stability analysis of *FOXC1* mutant polypeptides was carried out by transient expression of cDNA constructs encoding the different mutations in HEK-293T cells. The transfected cells were treated with cycloheximide, a protein synthesis inhibitor, and the recombinant proteins were detected by dot or Western immunoblot using an anti-myc monoclonal antibody (Santa Cruz Biotechnology) at the indicated time points. The transfection efficiency was assessed by co-transfection with the non-recombinant pMirTarget vector, which encodes RFP. RFP was detected by dot or Western blot using an anti-RFP antibody (Evrogen). The sample loading control, endogenous LDH, was also detected via immunoblot using an anti-LDH antibody (Chemicon). **(B)** The amounts of FOXC1 at the indicated time points were determined by densitometry of the corresponding signals obtained via western blot. The relative amounts of FOXC1 are expressed as a percentage of levels at time 0 h. **(C)** The rate of decay and half-lives of the recombinant FOXC1 variants at the indicated time-points were determined from linear regression analysis as described in the Materials and Methods. Error bars correspond to the SD of three independent experiments carried out in triplicate. insDG: p.G447_G448insDG. NT: non-transfected cells (negative control). Asterisks indicate statistical significance compared to the control: p<0.01 (**); p<0.001 (***). Two-way ANOVA followed by Tukey multiple-comparison test.

The protein stability of the FOXC1 mutants was assessed in transiently transfected cells treated with the protein synthesis inhibitor cycloheximide. The FOXC1 levels were evaluated using either dot blot or western blot at different times after the inhibition of the protein synthesis. Any significant transfection or loading sample variations were ruled out via a western blot analysis of RFP and LDH, respectively (Fig. 5A). p.I126S showed remarkably reduced stability (Fig. 5A and B), with an estimated half-life value that was approximately 3-fold lower than that of normal FOXC1 (4.35 h vs. 12.95 h, respectively, Fig. 5C), and almost identical to the half-life of the control mutation p.I126M (4.20 h, Fig. 5C). The dot-blot stability analyses of the two dominant nonsense mutations (p.Y47X and p.Q106X) showed the shortest half-lives among all of the mutants (< 2.4 h).

### p.I126S phosphorylation

**Fig 6:**
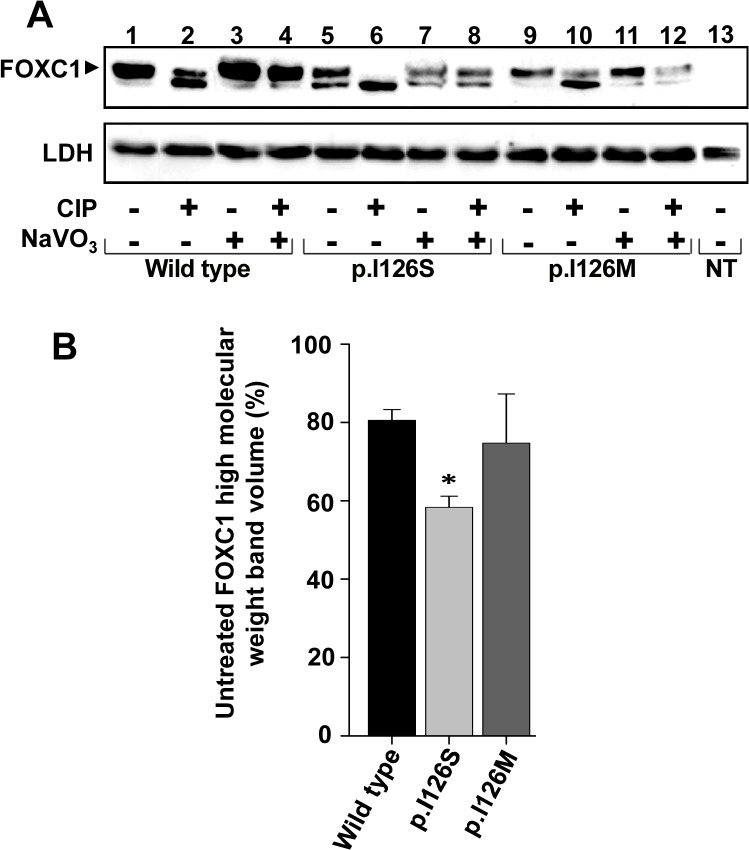
The p.I126S mutation decreases FOXC1 phosphorylation. **(A)** The nuclear extracts of HEK-293T cells transiently expressing the indicated FOXC1 mutants were treated with different combinations of CIP and sodium vanadate. After the treatments the recombinant proteins were detected via western immunoblot using an anti-myc antibody. The arrowhead indicates the position of the phosphorylated FOXC1. The sample loading control, endogenous LDH, was detected via immunoblot using an anti-LDH antibody (Chemicon). **(B)** The percentage of phosphorylated FOXC1 in untreated samples (CIP-, NaVO_3_-) was estimated by densitometry of the upper band as indicated in Materials and Methods. Error bars correspond to the SD of three independent experiments carried out in triplicate. NT: non-transfected cells (negative control). The asterisk indicates statistical significance compared to the control: p<0.05 (*); One-way ANOVA.

A careful western immunoblot analysis of FOXC1 revealed a doublet with a low molecular weight band that was more intense in p.I126S than in the wild-type protein (Fig. 6A, lanes 5 and 1, respectively). To assess whether this difference is due to phosphorylation, p.I126S was treated with CIP and analyzed via western blot. As controls, wild-type FOXC1 and the p.I126M mutant were treated in parallel. The LDH detection via western blot showed no significant sample loading variations (Fig. 6A). The untreated wild-type FOXC1 presented an upper band, estimated to represent 80% of the total protein (Fig. 6A, lane 1 and Fig. 6B). The proportion of this band in the untreated p.I126S (Fig. 6A, lane 5) represented less than 60% of the mutant protein (Fig. 6B), whereas in the control mutant was similar to the wild-type (Fig. 6A, lane 9 and Fig. 6B). The CIP treatment of all of the variants increased the lower band proportion (Fig. 6A, lanes 2, 6 and 10), and this effect was counteracted with the CIP inhibitor NaVO_3_ (Fig. 6A, lanes 3–4, 7–8 and 11–12), indicating that the upper band corresponds to phosphorylated FOXC1. In addition, the predicted phosphorylation potential of the mutant Ser residue indicated that it was unphosphorylated (data not shown). These results suggest that p.I126S can alter the FOXC1 conformation, decreasing the phosphorylation of the whole protein.

### EMSA assay

**Fig 7:**
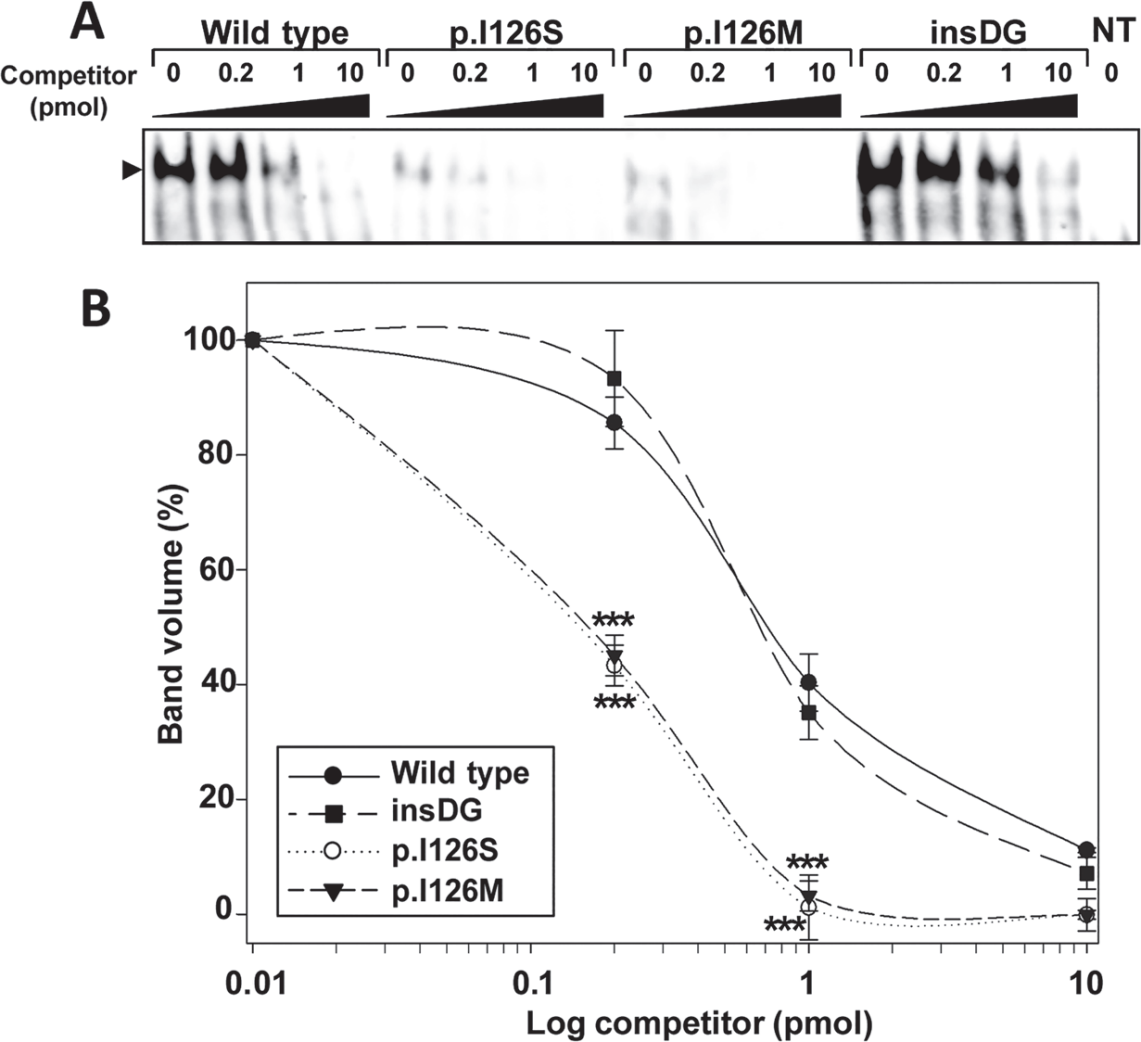
The p.I126S mutation decreases FOXC1 DNA-binding. **(A)** The effect of *FOXC1* mutations on DNA-binding specificity was assessed via EMSA. The oligonucleotides corresponding to the FOXC1-binding sequence labeled with biotin at the 5'-end were incubated with 10 μg of nuclear extracts from HEK-293T cells transfected with the indicated FOXC1 variants. The extracts were separated on a 10% nondenaturing polyacrylamide gel electrophoresis and were transferred to a positively charged nylon membrane (Hybond-N+, Amersham). FOXC1-oligonucleotide complexes were visualized by incubation with streptavidin-HRP conjugate and chemiluminescent detection (Chemiluminescent EMSA kit, Thermo Scientific). Unlabeled competitor oligonucleotides were pre-incubated at increasing concentrations from 0.2 to 10 pmol (1 to 50-fold excess) with the labeled probe. The arrowhead indicates the position of the full-length FOXC1-DNA complexes. **(B)** The percentage of band volume corresponding to protein-oligonucleotide complexes was estimated by densitometry as indicated in Materials and Methods. Error bars correspond to the SD of three independent experiments carried out in triplicate. insDG: p.G447_G448insDG. NT: non-transfected cells (negative control). Asterisks indicate statistical significance as compared to the control: p<0.001 (***). Two-way ANOVA followed by Tukey multiple-comparison test.

The DNA binding ability of the different mutations was evaluated using EMSA analysis. Competition experiments in which similar amounts of pre-bound protein-DNA complexes were challenged with increasing ratios of competitor DNA containing the FOXC1 binding site (ranging from 0.2 to 10 pmol, i.e., 1- to 50-fold excess), showed that both the intensity and the proportion of the protein-DNA complexes formed by the p.I126S and p.I126M (control) mutations, were significantly lower than those of the wild-type protein (Fig. 7A and B). Converesely, and although the intensity of the complexes corresponding to p.G447_G448insDG was higher than those of the normal protein (Fig. 7A), the DNA binding ability did not differ significantly from the wild-type (Fig. 7B). As expected, the two nonsense mutants failed to bind to the DNA because they lacked a complete forkhead domain (data not shown).

### Subcellular localization

**Fig 8:**
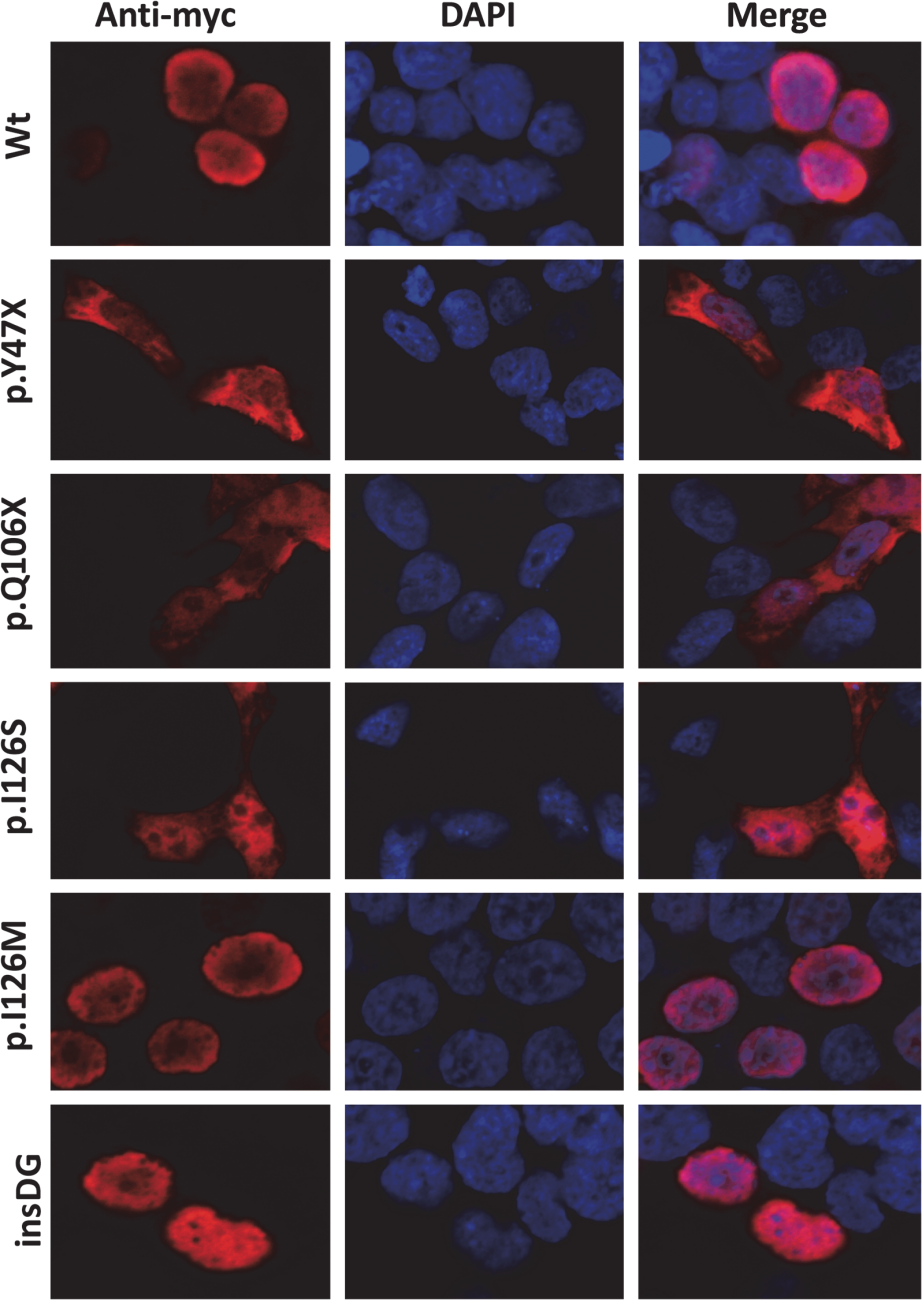
Altered subcellular localization of FOXC1 variants associated with dominat glaucoma. cDNA constructs encoding the indicated recombinant versions of FOXC1 were transiently expressed in HEK-293T cells. The recombinant proteins were detected via fluorescent immunocytochemistry using an anti-myc antibody (Santa Cruz Biotechnology). Nuclei were visualized using fluorescent DAPI staining. Original magnification: X600.

Fluorescence immunocytochemistry of the control recombinant wild-type FOXC1 localized the protein exclusively to the nucleus (Fig. 8, Wt). The two non-sense mutations were detected in both the nucleus and the cytoplasm (Fig. 8, p.Y47X and p.Q106X). Mutation p.I126S was also present in these two cell compartments. In contrast, the p.I126M control mutation was observed exclusively in the nucleus. p.G447_G448insDG also showed normal nuclear localization (Fig. 8).

## Discussion

Glaucoma is a genetically and clinically heterogeneous disease with a poorly understood genetic basis. It has been proposed that dominant glaucoma may originate from goniodysgenesis with marked variation in the expressivity of dysgenesis, leading to the apparition of symptoms early or late in life [1]. Typical congenital glaucoma, which is inherited as a non-dominant trait, is also considered to be part of the anterior segment dysgenesis (ASD) group of disorders because it originates via the maldevelopment of structures in the anterior segment of the eye. On the converse, *FOXC1* is a gene that regulates the development of the anterior segment of the eye, and it is known to be involved in several autosomal dominant eye defects associated with an increased risk for glaucoma. Its heterozygous loss-of-function in mice is associated with considerable variability in anterior segment abnormalities, similar to those present in patients with ARA and glaucoma [37]. Based on these facts, we explored the role of *FOXC1* in dominant glaucoma. To this end, we carried out a comprehensive screening of the coding and regulatory regions (UTRs and the promoter) of the gene in twelve affected Spanish families with no known alterations in other glaucoma genes.

### Pathogenicity of the *FOXC1* mutations

We identified three hypermorphic (p.Y47X, p.Q106X and p.G447_G448insDG) and one hypomorphic (p.I126S) heterozygous *FOXC1* variants in four probands, representing a significant proportion (33.3%) of the studied families. Only two of these mutations have previously been reported. p.Q106X was identified in a patient with an enucleated eye due to severe glaucoma and abnormalities of the anterior segment in the other eye [21]. Interestingly, p.Q106X-related mutations have been found to be associated with high phenotypic variability. For instance, p.Q120X was detected in patients with ARS and Peters’ anomaly in the same family [38], and p.Q123X has been identified in ARA patients and even in a normal subject [39].

It is accepted that *FOXC1* mutations are pathogenic and function by reducing the levels of protein, inhibiting DNA binding and/or reducing the levels of FOXC1 transactivation [22, 40]. To the best of our knowledge, this is the first report of hypermorphic *FOXC1* variants associated with dominant glaucoma, which agrees with previous studies showing a high glaucoma risk associated with increased gene dosage due to *FOXC1* gene duplication [23, 41, 42]. The unexpected hyperactivity of the two truncated proteins is likely due to the expression of the isolated activation domain 1, which appears to act as a potent and autonomous transcription up-regulator despite the reduced stability of the mutant polypeptides. According to these results it has been described that expression of N-terminal FOXC1 residues 1–30 and 1–65 present transactivation levels that are equivalent to or higher than the full-length FOXC1, and it has been suggested that the N-terminal regions are sufficient to activate transcription [20]. Because the truncated proteins also failed to bind DNA, and pull-down experiments indicated that they did not interact with wild-type FOXC1 (data not shown), we propose that the increased transactivation is a gain-of-function consisting in abnormal interactions with other transcription factors. Our findings complements the haploinsufficiency mechanism, which has frequently been invoked to underlie the pathogenicity of *FOXC1* non-sense alleles. The mislocalization of the truncated proteins (present in both the nucleus and the cytoplasm) is likely due to their small molecular size (< 50 kDa), which allows free diffusion through the nuclear pores. The remaining hypermorphic mutation, p.G447_G448insDG, did not differ from the wild-type protein in terms of the protein stability, DNA binding or subcellular localization. Nevertheless, its localization in a Gly-rich region, which is predicted to be unstructured (IDR4), may explain the observed hyperactivity. IDRs are known to lack a fixed tertiary structure and are present in different regulatory proteins, which include the transcription factors CREB [43] and p53 [44]. They participate in molecular interactions with other proteins or nucleic acids, and although they lack a defined structure when alone in solution, they undergo disorder-to-order transitions after binding to specific targets [45]. These data suggest that the increased transcription activity of p.G447_G448insDG may be also due to a gain-of-function that would result in altered binding to transcription factors, suggesting a possible role of the mutant amino acids in protein-protein interactions. p.I126S was hypomorphic and its decreased protein stability and DNA binding affinity were similar to that of the p.I126M control mutation. The reduction in DNA binding affinity may be due to structural alterations in the forkhead domain alpha-helix 3, which is predicted to play a role in major DNA groove recognition. Contrary to our results, p.I126M has been described to bind the FBE at near-wild-type FOXC1 levels [22]. This discrepancy may reflect different levels of excess unlabelled oligonucleotides used as competitors for FOXC1 DNA binding, which ranged from 1- to 50-fold in the present study and from 0.5- to 1.5-fold in a previous report. In contrast to p.I126M, p.I126S reduced FOXC1 phosphorylation and induced intracellular mislocalization of this protein. Because the amino acid residues involved in the p.I126M mutation are not phosphorylated, we postulate that a mutation-induced conformational change may interfere the interaction between kinases and FOXC1, leading to decreased FOXC1 phosphorylation. In accordance with our results, it has been described that I126 is critical for correct FOXC1 nuclear localization and transactivation, so that substitution of I126 for different amino acids leads to differentially disrupted nuclear localization and DNA binding [46]. Although the differences in protein phosphorylation and subcellular localization between these two mutations do not seem to correlate with the severity of the associated phenotypes, we should bear in mind that unidentified genetic modifiers may also contribute to the final phenotypic outcome.

### Phenotypic variability and residual *FOXC1* activity

**Fig 9:**
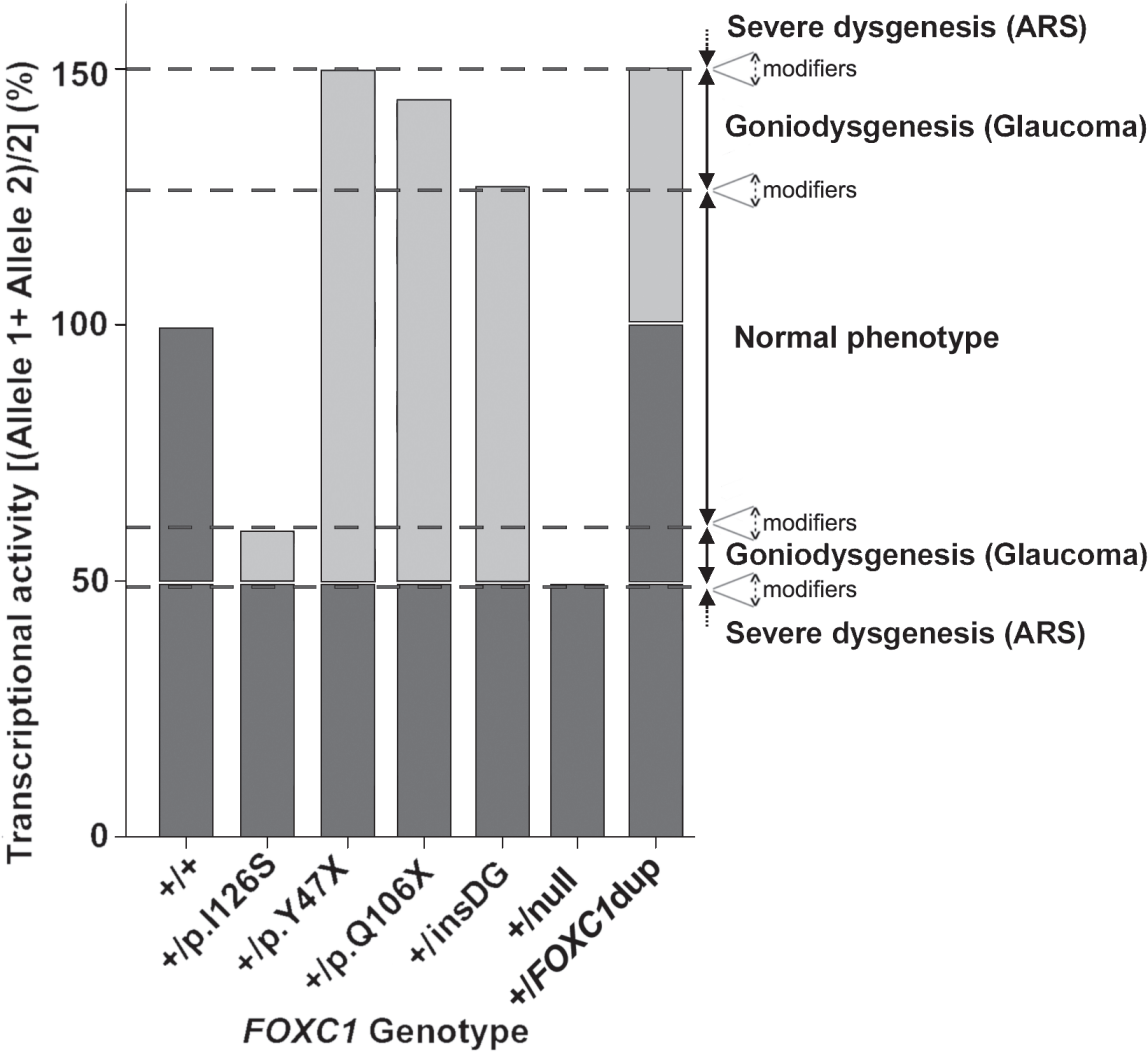
A model for *FOXC1* genotype-associated transcriptional activity and phenotypic variability. The transcriptional activity associated with each *FOXC1* genotype is calculated as the mean activity of the two *FOXC1* alleles. The model estimates that approximately ±35% activity variations are compatible with a normal ocular development. Mutant genotype activities ranging from approximately 50% to 60% or from 130% to 150% of the wild-type genotype, may lead to moderate goniodysgenesis and dominant glaucoma. Transactivation values equal to 150% [present in heterozygous *FOXC1* duplications (+-dup*FOXC1*)] or lower than 50% [associated with null *FOXC1* mutations (+-null) in the heterozygous state] are associated with anterior segment dysgenesis (ASD) and Axenfeld-Rieger syndrome (ARS). The different theoretical thresholds may change by influence of modifier genes and/or environmental factors (dashed arrows). Wild-type and mutant alleles are indicated in dark and light grey, respectively. insDG: p.G447_G448insDG.

Although dominant glaucoma was the most outstanding clinical feature in all of the mutation carriers, a variability in the age of onset was also observed. Moreover, one of the families (PCG73) also showed variable iris morphology, which ranged from thin stromal areas and posterior embrytoxon with no iris attachments (subjects II:2 and III:1), to corectopia associated with Axenfeld-Rieger anomaly (subject II:4). Mild iris alterations can be present in non-syndromic congenital glaucoma cases [47]. Previous reports have also identified a remarkable phenotypic variability associated with *FOXC1* mutations [39, 48]. This clinical heterogeneity suggests an important role for modifier factors (genetic, environmental and/or stochastic) on the phenotypic outcomes. The *FOXC1* associated phenotypic variability may also depend on the residual transcriptional activity associated with the individual genotypes. Based on this idea, we observed that none of the glaucoma-associated *FOXC1* mutations expressed in the cell cultures showed a complete loss-of-function. On the contrary, the glaucoma associated transcriptional activity ranged from roughly 20% to 198% of the wild-type levels. Using an additive model, we estimated the *FOXC1* transactivation ability associated with each glaucoma genotype as the mean activity of the two alleles and inferred two glaucoma associated activity intervals, which ranged from approximately 50% to 60% and from 130% to 150% compared to the wild-type *FOXC1* genotype (Fig. 9). Based on this hypothesis we postulate that the variation in the residual transactivation between these narrow limits primarily results in goniodisgenesis and leads to dominant glaucoma as the most important phenotypic feature. Moreover, FOXC1 activity levels beyond the upper and lower thresholds would lead to severe ASD affecting multiple ocular and even non-ocular tissues. This model also suggests that the normal ocular development tolerates only small FOXC1 activity fluctuations (approximately ± 35%). Consistent with this view, it has been proposed that *FOXC1* haploinsufficiency (50% of the normal activity) underlies dominant glaucoma in ARA patients [35] and increased gene dosage due to heterozygous gene duplications (150% activity, representing the upper activity value of this model compatible with alterations limited to the eye), causes dominant inherited anterior-chamber defects associated with developmental glaucoma [49]. We also postulate an important influence of modifier genes, environmental factors and/or stochastic events on these transactivation thresholds. This hypothesis agrees with the proposed existence of critical activity thresholds for FOXC1 and the related protein PITX2 [42].

## Conclusions

In summary, this study shows that *FOXC1* sequence variations with moderate activity defects are associated with dominant glaucoma and remarkable phenotypic variability. In addition, this is the first study to identify hypermorphic variants of this gene and will contribute to a better understanding of the genetic basis of dominant glaucoma, as well as the function of *FOXC1*.

## Supporting Information

S1 Fig Localization of primers used in this study to analyze *FOXC1*. The arrows show the position of the different PCR primers. Amplicons are indicated by horizontal brackets and the numbers between parentheses correspond to amplicon length. CR: coding region.
(TIF)

S2 Fig Tricine-SDS-PAGE electrophoresis of the truncated *FOXC1* mutations. To improve detection of the small molecular size mutants p.Y47X and p.Q106X, the nuclear extracts of HEK-293T cells transiently expressing these recombinant proteins were analyzed via Tricine-SDS-PAGE. The proteins were detected via western immunoblot using an anti-myc antibody. NT: non-transfected.
(TIF)

S1 Materials and Methods (DOCX)

S1 Table The primer sequences and PCR conditions used for *FOXC1* sequencing. (DOCX)

S2 Table The primer sequences and PCR conditions used for *FOXC1* site-directed mutagenesis. (DOCX)
