# Supplementary figures and images for: Hypo- and Hypermorphic FOXC1 Mutations in Dominant Glaucoma: Transactivation and Phenotypic Variability
Source: PLoS One. 2015 Mar 18;10(3):e0119272. doi: 10.1371/journal.pone.0119272 (PMC4364892; doi:10.1371/journal.pone.0119272)

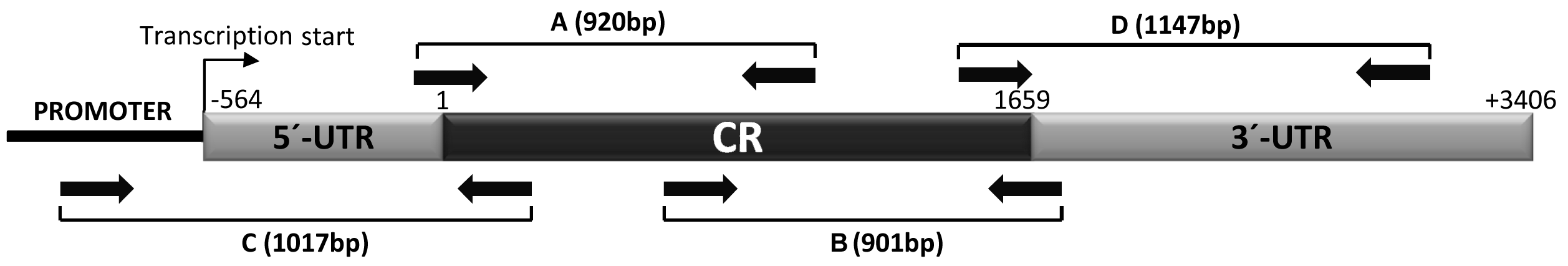

Supplement: S1 Fig — The arrows show the position of the different PCR primers. Amplicons are indicated by horizontal brackets and the numbers between parentheses correspond to amplicon length. CR: coding region. (TIF) [file pone.0119272.s001.tif]

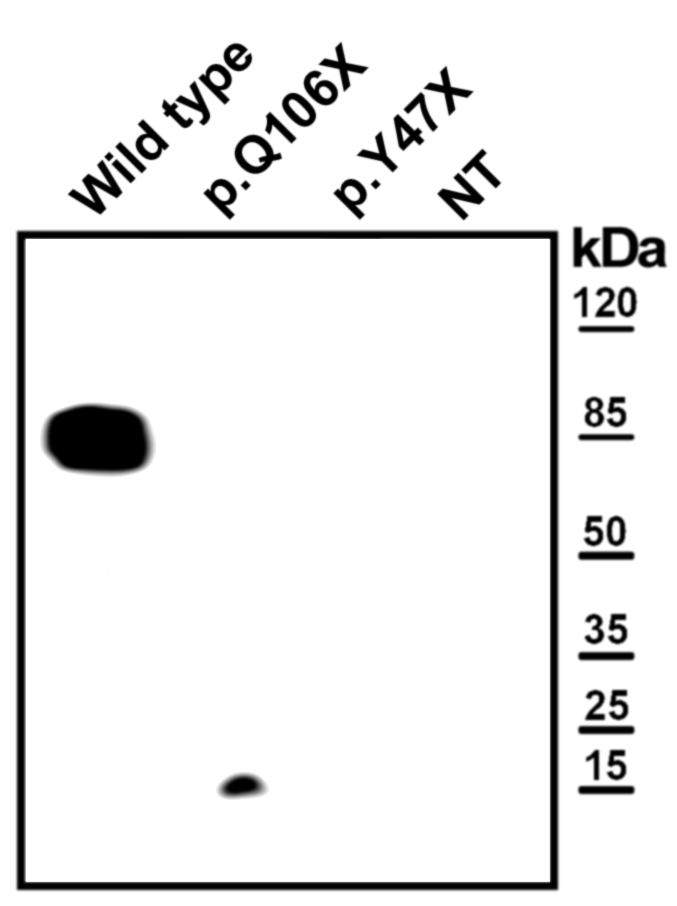

Supplement: S2 Fig — To improve detection of the small molecular size mutants p.Y47X and p.Q106X, the nuclear extracts of HEK-293T cells transiently expressing these recombinant proteins were analyzed via Tricine-SDS-PAGE. The proteins were detected via western immunoblot using an anti-myc antibody. NT: non-transfected. (TIF) [file pone.0119272.s002.tif]
