# Supplementary material for: Hypo- and Hypermorphic FOXC1 Mutations in Dominant Glaucoma: Transactivation and Phenotypic Variability
Source: PLoS One. 2015 Mar 18;10(3):e0119272. doi: 10.1371/journal.pone.0119272 (PMC4364892; doi:10.1371/journal.pone.0119272)
Supplement: S1 Materials and Methods — (DOCX) [file pone.0119272.s003.docx]

**Supplementary Materials and Methods**

**Site directed mutagenesis and cloning of *FOXC1* mutations**

**Three *FOXC1* mutations (**p.Y47X, p.Q106X and p.I126S) **identified in patients,** and the control p.I126M mutation**, was obtained by site-directed mutagenesis using** the QuickChange site-directed mutagenesis kit (Stratagene), according to the manufacturer's instructions. The primers and PCR conditions are indicated in S2 Table. The wild-type *FOXC1* coding sequence obtained from a commercial cDNA (Origene, NM_001453) was subcloned into the *Eco*RI and *Bam*HI restriction sites of the modified mammalian cell expression vector pcDNA3.1(-) (C-terminal Myc-Tagged) [1] by directional PCR using the following primers: 5’GG**GAATTC**GGGGCCATGCAGGCGCGCTACT3’ (P1) and 5’GT**GGATCC**CCAAACTTGCTACAGTCGTAGA3’ (P2) (*Eco*RI and *Bam*HI sequences are indicated in bold, respectively). This cDNA construct was used as a template for the site-directed mutagenesis of the coding mutations. All site-directed mutagenesis products were sequenced to confirm the presence of the mutation and were subcloned into either the *Eco*RI and *Bam*HI restriction sites of pcDNA3.1(-) or *Eco*RI and *Not*I of pMirTarget vector to avoid undesirable mutations in the vector. The variant p.G447_G448insDGwas directly amplified from the genomic DNA of one carrier using primers P1 and P2 and were cloned into the *Eco*RI/*Bam*HI restriction sites of the pcDNA3.1(-) vector.

**Culture of HEK-293T cells**

**The** cells were maintained in Dulbecco’s modified Eagle’s medium (DMEM) supplemented with 10% fetal bovine serum (FBS) and antibiotics (Normocin, Invitrogen) at 37°C in a fully humidified 5% CO_2_ atmosphere.

**Nuclear protein extraction and EMSA**

Forty-eight hours after transfection, cells were harvested, pelleted and resuspended in lysis buffer (20 mM HEPES pH 7.6, 150 mM NaCl, 0.5 mM DTT, 25% glycerol, 1.5 mM MgCl_2_, 2.5 mM phenylmethylsulfonyl fluoride, 1mM of NaVO_4_, 10 mg of Leupeptin/ml, and 1 mM of NaF) at 4ºC. Then cells were lysed by gentle sonication on ice and centrifuged at 13 000 *g* for 5 min at 4ºC, and the supernatants containing nuclear extracts were collected. To ensure that the same amount of protein was loaded in each lane, the protein content in nuclear extracts was determined by the Bicinchoninic Acid Protein Assay Kit (Thermo Scientific), following the manufacturer’s recommendations. EMSA was carried out using the nuclear extracts (10 µg protein) incubated with 1 µg poly[d(I-C)] in the binding buffer provided by the LightShift EMSA Optimisation and Control Kit (Thermo Scientific), and 0.2 pmol of the oligonucleotides containing the FOXC1-binding site (forward, 5'-GATCCAAAGTAAATAAACAACAGA3'; and reverse, 5'GATCTCTGTTGTTTATTTACTTTG3') for 15 min at room temperature. The forward oligonucleotide was labeled with biotin at the 5'- end. Bound DNA complexes were separated on a 10% nondenaturing polyacrylamide gel electrophoresis and transferred to a positively charged nylon membrane (Hybond-N+, Amersham). The membrane was UV cross-linked, probed with streptavidin-HRP conjugate and incubated with the chemiluminescent substrate for chemiluminescent detection (Chemiluminescent EMSA kit, Thermo Scientific). Unlabeled competitor oligonucleotides were pre-incubated at increasing concentrations from 1- to 50-fold excess with the labeled probe.

**Bioinformatic analyses**

The deleterious effect of mutations was predicted on line with the Sorting Intolerant From Tolerant (SIFT) [2], PolyPhen-2 (http://genetics.bwh.harvard.edu/pph2/) and Panther (http://www.pantherdb.org/tools/csnpScoreForm.jsp) programs. The Prosite database was searched to identify protein domains [3]. Both nucleotide and amino acid sequence alignments were carried out with Clustalw [4]. Disordered regions were predicted from the amino acid sequence using the DisEMBL (<http://dis.embl.de/>) [5] and Globplot 2 (http://globplot.embl.de/) [6] programs. The phosphorylation potential of FOXC1 mutations was predicted using the NetPhos 2.0 Server (<http://www.cbs.dtu.dk/services/NetPhos/>) [7].

**References**

1. Aroca-Aguilar JD, Sanchez-Sanchez F, Ghosh S, Coca-Prados M, Escribano J. Myocilin mutations causing glaucoma inhibit the intracellular endoproteolytic cleavage of myocilin between amino acids Arg226 and Ile227. 2005;J Biol Chem. 280: 21043-21051.

2. Kumar P, Henikoff S, Ng PC. Predicting the effects of coding non-synonymous variants on protein function using the SIFT algorithm. Nat Protoc. 2009;4: 1073-1081.

3. Sigrist CJ, de Castro E, Cerutti L, Cuche BA, Hulo N, Bridge A, et al. New and continuing developments at PROSITE. Nucleic Acids Res. 2013;41: D344-347.

4. Thompson JD, Higgins DG, Gibson TJ. CLUSTAL W: improving the sensitivity of progressive multiple sequence alignment through sequence weighting, position-specific gap penalties and weight matrix choice. Nucleic Acids Res. 1994;22: 4673-4680.

5. Linding R, Jensen LJ, Diella F, Bork P, Gibson TJ, Russell RB. Protein disorder prediction: implications for structural proteomics. Structure. 2003;11: 1453-1459.

6. Linding R, Russell RB, Neduva V, Gibson TJ. GlobPlot: Exploring protein sequences for globularity and disorder. Nucleic Acids Res. 2003;31: 3701-3708.

7. Blom N, Gammeltoft S, Brunak S. Sequence and structure-based prediction of eukaryotic protein phosphorylation sites. J Mol Biol. 1999. 294: 1351-1362.
