# Supplementary material for: Hypo- and Hypermorphic FOXC1 Mutations in Dominant Glaucoma: Transactivation and Phenotypic Variability
Source: PLoS One. 2015 Mar 18;10(3):e0119272. doi: 10.1371/journal.pone.0119272 (PMC4364892; doi:10.1371/journal.pone.0119272)
Supplement: S1 Table — (DOCX) [file pone.0119272.s004.docx]

| Table S1. The primer sequences and PCR conditions used for *FOXC1* sequencing. | | | |  |  |  |
| --- | --- | --- | --- | --- | --- | --- |
| **Primer set** | **Sequence (5’→3’)** | **Annealing temperature (ºC)/time (s)** | **PCR cycles** | | **Amplicon length (bp)** | |
| A | F: GGGAATTCGGGGCCATGCAGGCGCGCTACT | 55/120 | 43 | | 920 | |
|  | R: CCCTGGCTATGGTGCGG |  |  | |  | |
| B | F: GCCCAAGATCGAGAGCCC | 55/120 | 43 | | 901 | |
|  | R: GTGGATCCCCAAACTTGCTACAGTCGTAGA |  |  | |  | |
| C | F: GGAGGACCCAGGAAGTCTGC | 58/120 | 40 | | 1017 | |
|  | R: GTGCGAGTACACGCTCATGG |  |  | |  | |
| D | F: GAGTCACAGAGGATCGGCTTG | 57/120 | 42 | | 1147 | |
|  | R: AAATTTCCGAATCATGGACTGTC |  |  | |  |  |
| F: forward; R: reverse. | | | |  |  |  |
