# Supplementary material for: Hypo- and Hypermorphic FOXC1 Mutations in Dominant Glaucoma: Transactivation and Phenotypic Variability
Source: PLoS One. 2015 Mar 18;10(3):e0119272. doi: 10.1371/journal.pone.0119272 (PMC4364892; doi:10.1371/journal.pone.0119272)
Supplement: S2 Table — (DOCX) [file pone.0119272.s005.docx]

| Table S2. The primer sequences and PCR conditions used for *FOXC1* site-directed mutagenesis | | | | |
| --- | --- | --- | --- | --- |
| **Primer set** | **Sequence (5’→3’)** | | **Annealing temperature (ºC)/time (s)** | **PCR cycles** |
| p.Y47X | F: CCGGCCCCCATGAGCGTGTA**G**TCGCACCCTGCGC  R: GCGCAGGGTGCGA**C**TACACGCTCATGGGGGCCGG | 58/15 | | 30 |
| p.Q106X | F: CCCTGAACGGCATCTAC**T**AGTTCATCAT  R: ATGATGAACT**A**GTAGATGCCGTTCAGGG | 58/15 | | 30 |
| p.I126S | F: GCTGGCAGAACAGCA**G**CCGCCACAACCTCTCG  R: CGAGAGGTTGTGGCGG**C**TGCTGTTCTGCCAGC | 58/15 | | 30 |
| p.I126M | F: TGGCAGAACAGCAT**G**CGCCACAACCTCTC  R:GAGAGGTTGTGGCG**C**ATGCTGTTCTGCCA | 58/15 | | 30 |
| F: forward; R: reverse. The mutant nucleotides are indicated in bold. | | | | |
